# Quercetin: A Treatment for Hypertension?—A Review of Efficacy and Mechanisms

**DOI:** 10.3390/ph3010237

**Published:** 2010-01-19

**Authors:** Abigail J. Larson, J. David Symons, Thunder Jalili

**Affiliations:** 1 Department of Exercise and Sport Science, University of Utah, HPER North, 250 South 1850 East, SLC UT, 84112, USA; E-Mails: ab.larson@utah.edu (A.J.L.); J.David.Symons@hsc.utah.edu (J.D.S.); 2 Department of Nutrition, University of Utah, HPER North, 250 South 1850 East, SLC UT 84112, USA

**Keywords:** quercetin, hypertension, angiotensin converting enzyme, endothelial function, antioxidants

## Abstract

Quercetin is a polyphenolic flavonoid. Common sources in the diet are apples, onions, berries, and red wine. Epidemiological studies have found an inverse relationship between dietary quercetin intake and cardiovascular disease. This has led to *in vitro*, *in vivo*, and clinical research to determine the mechanism by which quercetin exerts cardio-protective effects. Recent studies have found a reduction in blood pressure when hypertensive (>140 mm Hg systolic and >90 mm Hg diastolic) animals and humans are supplemented with quercetin. Proposed mechanisms for the antihypertensive effect of quercetin include decreased oxidative stress, inhibition of angiotensin converting enzyme activity, improved endothelial function, direct action on the vascular smooth muscle, and/or modulation in cell signaling and gene expression. Although *in vitro* and *in vivo* evidence exists to support and refute each possibility, it is likely that quercetin influences multiple targets via a combination of known and as yet undiscovered mechanisms. The purpose of this review is to examine the mechanisms whereby quercetin might reduce blood pressure in hypertensive individuals.

## 1. Introduction

The American Heart Association estimates that 80 million Americans currently suffer from essential hypertension [1]. The pathogenesis and pathophysiology of hypertension is a complex heterogeneous disorder that can co-exist with other cardiovascular and metabolic abnormalities. It is defined as systolic blood pressure consistently over 140 mm Hg and diastolic blood pressure over 90 mm Hg and represents persistently elevated vascular resistance, yet hypertension is usually asymptomatic [1]. 

Blood pressure (BP) is controlled by neural and humoral mechanisms. Neural regulation is carried out by the autonomic nervous system whereas humoral regulation is performed by a variety of substances released by different cell types [2]. Hypertension is largely due to inappropriate humoral control [2] precipitated by increased oxidative stress, increased production of endothelin-1 (ET-1), decreased nitric oxide (NO) production and/or bioavailability, and/or over-stimulation of the renin-angiotensin system (RAS). Moreover, these changes have been associated cardiovascular disease (CVD) [2,3,4,5,6,7,8] and there is a positive and direct correlation between hypertension and risk for cardiac arrhythmia, cardiac hypertrophy, myocardial infarction, and heart failure [1,9]. 

Treatment of hypertension depends on the etiology of the disease and includes diet alterations, weight loss, exercise, and pharmacological interventions. Pharmacological therapies [e.g., angiotensin converting enzyme (ACE) inhibition, diuretics, and calcium channel blockers] that exist to treat hypertension are successful but may be associated with negative side effects such as persistent cough, dry throat, allergic reactions, dizziness, angioedema, and kidney failure [10]. Lifestyle interventions such as reduced sodium intake, the American Heart Association DASH (Dietary Interventions to Stop Hypertension) Diet, and increased physical activity are also known to lessen the severity of hypertension [1]. In addition to the aforementioned traditional therapies, dietary supplements, also known as nutraceuticals, are becoming increasingly popular in the treatment and prevention of hypertension. Increased supplement use, in general, to treat medical conditions may be due to an inability to maintain lifestyle changes as well as a desire to decrease use of pharmaceuticals; however, many supplements have unknown efficacy, safety, and mechanisms of action. Recently there has been an abundance of research examining dietary phytochemical supplements, such as quercetin, and their effect(s) on vascular health. The purpose of this review is to examine mechanisms by which supplemental quercetin reduces BP in hypertensive individuals.

## 2. Quercetin

Phytochemicals, or phytonutrients, are biologically active plant constituents that occur naturally to protect against insect invasion, disease, and infection. Phytochemicals also provide color, flavor, and aroma. Flavonoids are a specific class of phytochemicals which are divided into more than 10 different subclasses based on their molecular structure. The structure of the flavonoid also determines particular contributions to the health benefits associated with fruit and vegetable consumption [11]. Quercetin is a phytochemical belonging to the flavonoid family and is the most ubiquitous of the dietary flavonoids. Apples and onions are primary sources in the western diet; other foods containing quercetin include citrus fruits, berries, red grapes, red wine, broccoli, bark roots, flowers, and tea (Table 1). The average western diet supplies 15–40 mg of quercetin a day and higher dietary levels (> 33 mg/day) have been associated with decreased risk of CVD [12,13,14]. Because of the high variability in flavonoid content of any given food due to soil, harvest, and storage conditions; quantifying dietary quercetin intake precisely is extremely difficult [15,16]. Quercetin is also available over the counter in the form of supplements that can contain up to 250–1,500 mg quercetin. These large doses are used to homeopathically “treat” a variety of ailments such as allergies, asthma, bacterial infections, arthritis, gout, eye disorders, hypertension, and neurodegenerative disorders. Because few controlled randomized trials have been performed, little data exist to provide a solid scientific basis for these treatment claims.

**Table 1:** Amount of quercetin in selected foods, adapted from the USDA database [17].

Food	Quercetin Content
Capers	233 mg/100 g
Onions	22.0 mg/100 g
Cocoa powder	20.0 mg/100 g
Cranberries	14.0 mg/100 g
Lingonberries	7.4 mg/100 g
Apples	4.57 mg/100 g
Green tea	2.69 mg/100g
Black tea	1.99 mg/100g
Catsup	0.86 mg/100 g

### Bioavailability of Quercetin

**Figure 1:**
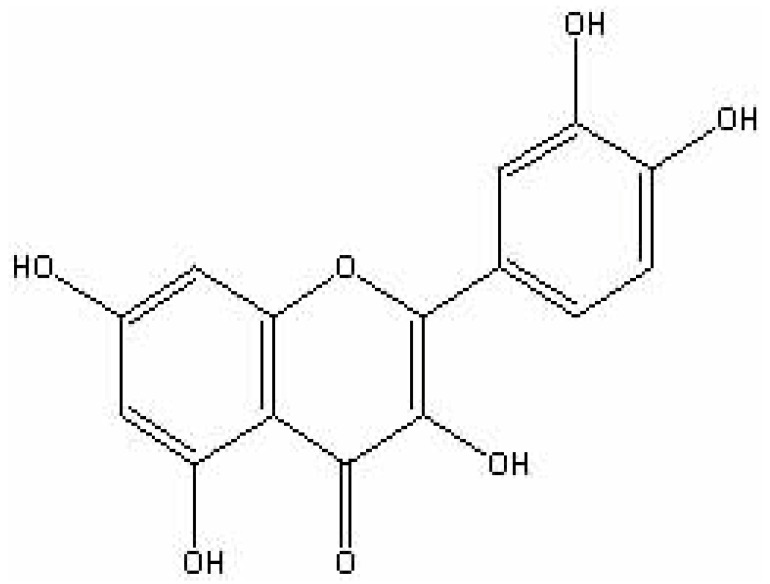
Quercetin aglycone.

**Figure 2:**
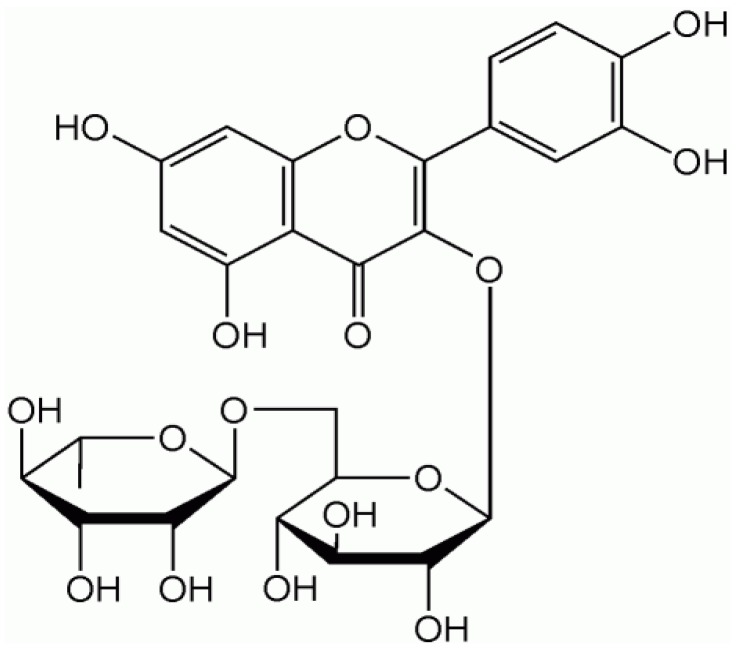
Conjugated quercetin (rutin).

The bioavailability of quercetin depends on the absorption and metabolism of this flavonoid. Absorption is dependent upon the type ingested (e.g., quercetin aglycone or quercetin glycosides), the food matrix in which it is found, and individual differences in colon flora [15,16,18,19,20,21,22,23,24]. The chemical structure of pure quercetin is an un-conjugated aglycone (Figure 1). Quercetin glycosides (Figure 2) have a carbohydrate moiety and are more commonly found in foods [25]. Supplements are sold in both forms and studies indicate that both are readily bioavailable [18,19,22,23,24,26]. We have found that healthy males given a single dose of 1,095 mg quercetin aglycone show a significant increase, from baseline, in plasma quercetin and quercetin metabolite concentration after only 3 hours (0.37 ± 0.04 μM *vs*. 1.05 ± 0.16 μM P < 0.05). Additionally, we observed that plasma concentrations were maximal (2.33 ± 0.72 μM) 10 hours after ingestion, and returned to baseline within 24 hours (0.45 ± 0.06 μM) (P < 0.05) [27].

Quercetin is absorbed in the small intestine and colon where it and its glycosides are conjugated with glucuronic acid in the intestinal epithelium. It is then bound to albumin and transported to the liver [11]. Very little free quercetin is found in circulation, and most appears in the blood in glucuronide form and as quercetin metabolites (isorhamnetin and kaempferol) [21]. Despite the potential physiological significance of quercetin in its metabolized form, much in vitro research has examined quercetin in a form that would not be present *in vivo* (see “Direct action on the VSM”). 

## 3. Quercetin: A Treatment for Hypertension?

### 3.1. Effects of Quercetin on BP in Animals

Our laboratory and others have reported that quercetin decreases BP and/or reduces the severity of hypertension in spontaneously hypertensive rats (SHR) [28,29], rats fed a high-fat high-sucrose diet [30], rats deficient in NO [31], rats infused with angiotensin [32], rats with aortic constriction [33], and Dahl salt sensitive rats [34]. Quercetin has also been shown to have in vitro vasodilator effects in isolated rat arteries [35,36]. While translating results observed in animals directly to humans should be done with caution, these studies nevertheless provide proof of principle that a quercetin-induced reduction in BP might be responsible for the reduction of CVD risk observed in humans with high quercetin diets.

### 3.2. Effects of Quercetin on BP in Humans

Epidemiological studies have found an inverse relationship with flavonoid intake and chronic disease [12,13,14]. Evidence from the Zutphen Elderly Study [13] suggests a strong cardio-protective effect of several flavonoids, including quercetin. In this study the risk of coronary death was reduced by as much as 68% in men who consumed >29 mg flavonols/day compared to men who consumed <10 mg flavonols/day. While the specific association between quercetin intake and BP was not examined in this study, the authors did report an inverse relationship between high quercetin-containing foods and BP [13].

**Figure 3:**
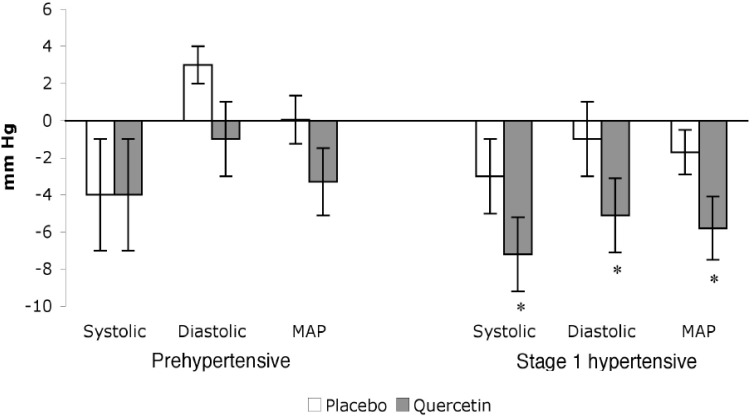
Reduction of BP from baseline in quercetin and placebo treated subjects. Data adapted from Edwards *et al.* [37]**.**

Supplementation of the diet with quercetin has been shown to reduce BP in hypertensive individuals. We conducted a randomized, double-blind, placebo-controlled crossover trial using stage 1 hypertensive (n = 22) and prehypertensive (n = 19) participants to investigate the efficacy of quercetin supplements. Reductions (p < 0.01) in systolic (–7 ± 2 mm Hg), diastolic (–5 ± 2 mm Hg), and mean arterial BP (–5 ± 2 mm Hg) were observed in stage 1 hypertensive men and women after supplementation with 730 mg quercetin/ day for 28 days *vs*. placebo treatment (Figure 3). Reductions of this magnitude are clinically relevant and associated with a 14% and 9% decrease in mortality from stroke and coronary heart disease, respectively [1]. In this study, quercetin did not evoke BP reductions in pre-hypertensive individuals (n = 19) (Figure 3) [37]. This latter finding agrees with other studies that report quercetin does not lower BP in pre-hypertensive or normotensive individuals or animal models [27,29,31,38]. The ability of quercetin to lower BP in hypertensive but not prehypertensive or normotensive models is not unique to this flavonoid. For example, the BP lowering effect of a diet rich in fruits and vegetables in hypertensive patients is well known; however, no reduction in BP is observed in normotensive subjects after a similar dietary intervention [39]. 

In contrast to the aforementioned studies, a recent study by Egert *et al.*, [40] found quercetin reduced BP in overweight and obese prehypertensive ( >120–139 mm Hg systolic and >80–89 mm Hg diastolic) individuals (N = 93); SBP was reduced by 2.9 ± 9.5 mm Hg after 150 mg/day quercetin for 6 weeks (P < 0.05). Discrepancies in the effect of quercetin on prehypertensives in the study by Egert *et al.* and our study, which reported no reduction in BP in prehypertensives, might result from the longer duration of supplementation (e.g., 42 d *vs*. 28 d) and / or the greater number of subjects (n = 93 *vs*. 19), respectively. With regard to the latter point, a larger sample size increases statistical power and thus the ability to detect smaller changes in BP.

## 4. Potential Mechanisms for Blood Pressure Reduction

Evidence exists to support several potential mechanisms whereby quercetin might reduce BP and decrease the severity of hypertension in animals and humans. For example, quercetin might reduce oxidative stress, interfere with the RAS, and /or improve endothelial and/or vascular function.

### 4.1. Oxidative Stress

In the past, the mechanism for quercetin-induced BP reduction in hypertensive animals and humans has been attributed to a reduction in oxidative stress. Animal studies that have observed reduced BP after quercetin supplementation have also shown improvements in oxidant status, such as reduction in plasma lipid peroxides [29,30,31] and urinary isoprostanes [29] when compared to untreated animals. It was hypothesized that improvement in oxidant status was the underlying mechanism behind improved vascular function observed in these studies [29,30,31].

In contrast, human investigations, have not clearly and consistently demonstrated an antioxidant effect of quercetin, even when using relatively high doses [19,26,37]. For example, Egert *et al*. [19], demonstrated that 2 weeks of quercetin supplementation in healthy, normotensive individuals did not affect plasma oxidized LDL, ferric reducing antioxidant potential (FRAP), or oxygen radical absorbance capacity (ORAC). Likewise, we have found similar results after 4 weeks of 730 mg/day quercetin supplementation in pre-hypertensive and stage 1 hypertensive individuals [37]. In our study we observed similar levels of plasma antioxidant capacity and urinary isoprostane concentration in the quercetin and placebo treatment, regardless of any change in blood pressure evoked by quercetin supplementation [37]. In contrast, a later study by Egert *et al.* [38] examined obese and overweight individuals with metabolic syndrome found reductions in BP accompanied by lower oxidized LDL after 6 weeks of daily quercetin supplementation when compared to baseline (p < 0.001) and placebo values (p < 0.05). However, there was no difference in plasma antioxidant capacity. 

While a great deal of evidence obtained from hypertensive animal models indicates that quercetin might be effective in lowering oxidant load, available data from humans is equivocal. In general, higher doses of quercetin have been evaluated in animals *vs*. humans, and this may lead to significant differences in the intracellular concentrations of quercetin, and subsequent antioxidant effects. For example, we have previously observed reductions in liver malondialdehyde levels in quercetin supplemented rats (150 mg quercetin/kg) [33], but no change plasma antioxidant power or urinary isoprostanes in quercetin supplemented humans (~8.1 mg quercetin/kg) [37]. In these studies we achieved plasma quercetin-metabolite levels 3.96 μg/mL in rats, but only 0.48 μg/mL in humans. Therefore it is possible that the disparate effects on oxidative stress were due to the dramatic difference in tissue / cellular quercetin concentrations. Given the inconsistent evidence regarding quercetin’s antioxidant effects, it is possible that the BP lowering effect of quercetin may be due to other more dominant mechanisms. However, further investigation of the antioxidant capacity of quercetin and its metabolites in humans may elucidate whether or not a localized antioxidant effect (e.g., at the endothelium) may play a role in BP reduction.

### 4.2. Rennin-Angiotensin System

**Figure 4:**
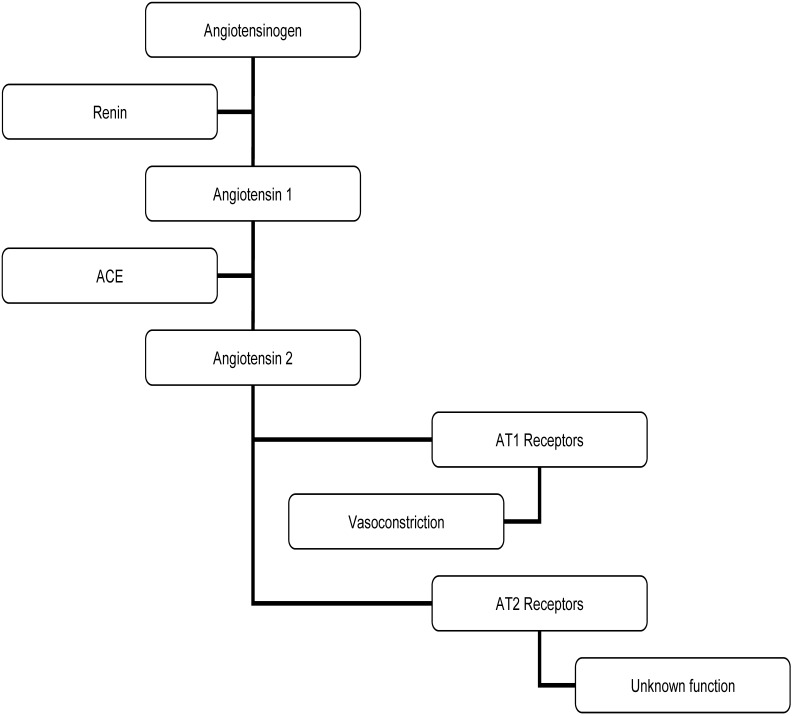
The renin-angiotensin system (RAS).

The RAS (Figure 4) is involved in the regulation of BP and controls fluid loss. Long-term over-activation of the RAS is associated with hypertension and can have negative cardiovascular effects [9]. Interference with the RAS by pharmacological ACE inhibitors such as captopril and imidapril can reduce circulating angiotensin II, a potent vasoconstrictor, resulting in lower blood pressure and fewer cardiovascular events in high-risk populations [1,10]. Pharmacological inhibitors, such as captopril and imidapril, inactivate the ACE molecule via binding a zinc molecule at the active site and slow the conversion of angiotensin I to angiotensin II [5,41]. Some flavonoids are also known to bind metal ions, such as zinc. Quercetin is particularly known for this property [36] and there is evidence that quercetin may inhibit ACE activity via this mechanism [42].

The hypothesis that quercetin might reduce BP *via* ACE inhibition/attenuation has been tested in animal models. Hackle *et al.* [32] examined the acute effects of oral and intravenous (IV) quercetin and captopril administration on the BP responses to bradykinin and angiotensin 1 infusion in normotensive Wistar rats. Bradykinin-evoked hypotension was potentiated by captopril and quercetin but not placebo treatment. Moreover, angiotensin 1-induced hypertension was blunted by captopril and quercetin but not placebo treatment (p < 0.05). In this study authors reported a 31% decrease in ACE activity after quercetin treatment *vs*. baseline [32], suggesting that quercetin acted as an ACE inhibitor. While this is a significant finding, it is difficult to determine the relative “strength” of quercetin’s ACE inhibitory effect since there was no direct comparison made against captopril evoked ACE inhibition in this study. 

Mackraj *et al.* [34] also compared the antihypertensive effects of captopril to quercetin using Dahl salt sensitive rats which were separated into three groups and injected daily for 4 weeks with captopril, quercetin, or placebo. BP decreased similarly in captopril and quercetin treated groups and increased in the untreated control group (p < 0.05). Captopril and quercetin treated groups also showed down-regulation of the AT1 receptor in the kidney when compared to the untreated group (p < 0.05) [34]. Taken together, these data indicate that chronic quercetin supplementation might reduce BP in a salt sensitive model of hypertension through modulation of renal function and AT1 receptor transcription. However, because ACE activity was not assessed in this study it is unclear whether quercetin acted as an ACE inhibitor as well. The mechanism for BP reduction is likely to have been a result of down-regulation of AT1a expression in the kidney. While this mechanism may explain the hypotensive effect of chronic quercetin supplementation, it is unlikely to explain the acute reductions in BP seen by Hackle *et al.*

Given the evidence in animals that indicates quercetin may act as an ACE inhibitor and can reduce AT1a expression, it is possible that similar mechanisms might be responsible for reductions in BP observed in human studies. However, no published research exists regarding either possibility. In light of the proven efficacy of pharmacological ACE inhibitors in humans, there is a need to conduct clinical trials to determine whether quercetin-induced decreases in ACE activity might be responsible for the BP lowering effects this flavonoid exerts in hypertensive humans.

### 4.3. Vascular Function

Endothelial cells line the walls of the arterioles and play a very important role in maintaining vascular homeostasis, vascular tone, cardiovascular and microvascular health. Endothelial dysfunction is a critical event in the pathogenesis of CVD and an independent predictor of cardiovascular events [43]. It is a common feature in all forms of CVD, including hypertension [43,44]. The endothelium releases a variety of vasoactive substances such as nitric oxide (NO) and endothelin-1 (ET-1), which serve, in part, to regulate BP and blood flow [2,4,43,45,46]. A common feature of endothelial dysfunction is diminished bioavailability of the vasodilator NO which results in less endogenous opposition to circulating vasoconstrictors such as ET-1 [4]. NO and ET-1 appear to have a reciprocal regulation; as NO bioavailability decreases there is enhanced synthesis of ET-1 [4]. Endothelial dysfunction is reversible and interventions that reduce risk for CVD, such as exercise and pharmaceuticals, can also improve endothelial function [4]. For this reason, it has become a surrogate biomarker and endpoint for determining efficacy of CVD interventions [4,47]. 

Endothelium-dependent vasorelaxation and proper endothelial function is largely dependent on the production and bioavailability of NO. Due to its short half-life NO is very difficult to assess *in vivo*. Indirect measurements of NO, such as flow mediated dilation (FMD) and concentration of NO metabolites (nitrites and nitrates) can be used to estimate endogenous production [26,30]. FMD in the peripheral circulation is primarily mediated by endothelium-derived NO in response to increased flow and shear stress, and results in smooth muscle relaxation and arterial dilation [4,46]. 

Previous studies using animal models have shown that quercetin induced reductions in BPare accompanied by** improvements in endothelial function and biomarkers of endothelial function [29,30,31,36]. For example, Duarte *et al.* [29] found that quercetin reduced BP in SHR, and improved endothelium-dependent vasorelaxation of isolated aorta. In another study, Duarte *et al.* [31] administered the NO synthase (NOS) inhibitor, L-NAME, to the drinking water of rats causing them to become hypertensive. Rats treated with quercetin exhibited less severe L-NAME-induced hypertension and endothelium-dependent dysfunction of arteries. Similarly, Yamamoto *et al.* [30] used a dietary model of hypertension (high-fat, high-sucrose diet for 4-weeks), and reported that systemic hypertension, reduced aortic NOS activity, and decreased urinary NO metabolites were ameliorated in rats that concurrently consumed quercetin. Taken together these studies indicate that quercetin can improve endothelial function via increasing NO bioavailability and/or NO production, and that these improvements may be responsible for reduction of BP. 

A recent study by Loke *et al.* [26], conducted in humans, reported that quercetin has the potential to improve the balance between circulating ET-1 and NO. Healthy, normotensive males were given a single 200 mg dose of quercetin or placebo; after 2 hours, plasma ET-1 was lower and after 5 h urinary metabolites of NO were higher in quercetin *vs*. placebo treatment. There were no changes in measurements of oxidative stress. While BP was not measured in this study, these data provide proof of principle that quercetin has potential to beneficially alter endothelial function and vascular tone. 

FMD in humans has been examined after the chronic and acute consumption of foods and beverages very high in flavonoids. In a placebo-controlled, crossover study, using participants with coronary artery disease (n = 50), Vita *et al.* [47] found acute and chronic tea consumption improved FMD, without an effect on nitroglycerin-mediated dilation, compared to placebo (P < 0.05). Because FMD was not influenced by an acute dose of caffeine, improvements were not secondary to this component of tea. No markers of oxidative stress were altered, suggesting that improvements were not due to an antioxidant effect [47]. Since BP was not assessed in this study it is unclear whether FMD improvements resulted in beneficial hemodynamic alterations. Given that tea contains an assortment of polyphenolic compounds, including quercetin, it is difficult to ascertain if the improvement in endothelial function was due to a single dominant ingredient, such as quercetin, or due to the synergistic action of combined polyphenolic compounds. These findings are similar to other studies that have examined the benefits of flavonoid-rich foods on endothelial function [25]. Although quercetin is an abundant flavonoid and present in the foods and beverages examined in the previous studies, changes in FMD solely as a result of acute or chronic quercetin administration have not yet been assessed. Likewise, it is unknown whether quercetin-induced improvements in endothelial function—should they occur—will result in a reduction of BP in humans.

There is also evidence that supports a role for quercetin to alter gene expression [3,34,48,49,50]. Nicholson *et al.* [48] investigated the effects of quercetin on gene expression in human umbilical vein endothelial cells (HUVEC) and found quercetin down-regulated ET-1 expression significantly. Zhao *et al.* [50] also found a dose-dependent decrease in ET-1 release after *ex vivo* administration of quercetin in HUVEC. These findings provide support for the hypothesis that quercetin might reduce ET-1 and thereby improve vascular function by altering the balance between circulating constrictor and dilator substances. Additional research is needed to confirm that these findings translate to humans *in vivo*. 

### 4.4. Direct Action on the Vascular Smooth Muscle

There is also evidence that quercetin may reduce BP through mechanisms independent of the endothelium. For example, quercetin has been shown to evoke vasorelaxation in endothelial-denuded vessels, suggesting that this flavonoid can act directly on the vascular smooth muscle (VSM) [35,36,51]. In addition to the parent flavonoid, metabolites of quercetin also might act directly on VSM. Perez-Vizcaino *et al.* [35] reported quercetin and isorhamnetin (quercetin metabolite) induced vasorelaxation of rat aortic rings similarly regardless of whether the endothelial layer of vessels was intact or denuded. Likewise, Rendig *et al.* [51] found that the enhanced vasorelaxation in resistance and conductance rabbit vessels after quercetin treatment was independent of the presence of the endothelium. It should be noted that since these studies used normotensive animals and did not assess BP, a comparison against previously mentioned findings is difficult. How quercetin acts on the VSM is not clear, but it has been speculated that vasodilation may result from inhibition of protein kinases involved in the Ca^2+^ sensitizing mechanisms responsible for smooth muscle contraction [35]. The effect of quercetin on the VSM of humans is unclear. FMD studies indicate that foods high in quercetin improve endothelium-dependent vasodilation but do not effect endothelium-independent dilation [47]; however, this has yet to be examined in trials involving quercetin supplementation.

## 5. Safety of Quercetin

Quercetin is believed to be antimutagenic *in vivo* and long-term studies have found that quercetin is not carcinogenic [52]. Very few negative side effects have been noted with short-term (<3 months), high intakes of quercetin [19,37,38], but there have been reports of nausea, headache, and tingling of the extremities with chronic quercetin supplementation of 1,000 mg/day (as reviewed by Harwood *et al.* [52]). Our studies to date with humans, including research in progress, have not found any adverse side effects with chronic doses of 730 mg/day for 28 days, or an acute dose of 1,095 mg quercetin aglycone in humans [37]. Quercetin is also an inhibitor of CYP3A4, an enzyme that breaks down many commonly prescribed drugs in the body; therefore, quercetin should not be taken with drugs that depend on this enzyme for metabolism. Since many flavonoids have been found to inhibit platelet aggregation (via inhibition of thromboxane A2) [3,47], it is also possible that pharmacological doses of quercetin could increase risk of bleeding when taken with anticoagulant drugs. 

## 6. Conclusions

Despite the uncertainty of quercetin’s mechanism of action of BP reduction in humans, it holds promise for the treatment of hypertension and promoting cardiovascular health. *In vitro* and *in vivo* research in animal models has shown multiple mechanisms of action that could produce the BP lowering effect seen in hypertensive humans. In conjunction with identifying primary mechanisms, more controlled randomized human research studies are needed to confirm quercetin’s efficacy, magnitude of effect, and optimal dosing schedule. Furthermore, it needs to be determined if quercetin is an effective treatment for all forms of hypertension regardless of pathological origin. Studies including different ethnicities and women are still scarce and long-term studies examining the safety of quercetin supplementation need to be conducted before recommending this flavonoid as a treatment option for the public. 
